# Receptor-Induced Dilatation in the Systemic and Intrarenal Adaptation to Pregnancy in Rats

**DOI:** 10.1371/journal.pone.0004845

**Published:** 2009-03-16

**Authors:** Vanessa M. Ferreira, Thiago S. Gomes, Luciana A. Reis, Alice T. Ferreira, Clara V. Razvickas, Nestor Schor, Mirian A. Boim

**Affiliations:** 1 Department of Medicine, Renal Division, Federal University of São Paulo, São Paulo, São Paulo, Brazil; 2 Department of Pathology, Federal University of São Paulo, São Paulo, São Paulo, Brazil; 3 Department of Biophysics, Federal University of São Paulo, São Paulo, São Paulo, Brazil; Universidad Europea de Madrid, Spain

## Abstract

Normal pregnancy is associated with systemic and intrarenal vasodilatation resulting in an increased glomerular filtration rate. This adaptive response occurs in spite of elevated circulating levels of angiotensin II (Ang II). In the present study, we evaluated the potential mechanisms responsible for this adaptation. The reactivity of the mesangial cells (MCs) cultured from 14-day-pregnant rats to Ang II was measured through changes in the intracellular calcium concentration ([Cai]). The expression levels of inducible nitric oxide synthase (iNOS), the Ang II-induced vasodilatation receptor AT2, and the relaxin (LGR7) receptor were evaluated in cultured MCs and in the aorta, renal artery and kidney cortex by real time-PCR. The intrarenal distribution of LGR7 was further analyzed by immunohistochemistry. The MCs displayed a relative insensitivity to Ang II, which was paralleled by an impressive increase in the expression level of iNOS, AT2 and LGR7. These results suggest that the MCs also adapt to the pregnancy, thereby contributing to the maintenance of the glomerular surface area even in the presence of high levels of Ang II. The mRNA expression levels of AT2 and LGR7 also increased in the aorta, renal artery and kidney of the pregnant animals, whereas the expression of the AT1 did not significantly change. This further suggests a role of these vasodilatation-induced receptors in the systemic and intrarenal adaptation during pregnancy. LGR7 was localized in the glomeruli and on the apical membrane of the tubular cells, with stronger labeling in the kidneys of pregnant rats. These results suggest a role of iNOS, AT2, and LGR7 in the systemic vasodilatation and intrarenal adaptation to pregnancy and also suggest a pivotal role for relaxin in the tubular function during gestation.

## Introduction

The maternal adaptation to pregnancy includes a reduced vascular reaction to vasoconstrictors, which results in systemic and intrarenal vasodilatation followed by increases in the renal plasma flow and the glomerular filtration rate [1], [2]. Pregnancy-associated vasodilatation has been attributed, in part, to hormones such as prostaglandins [3], nitric oxide (NO) [3], [4], [5], endothelium-derived hyperpolarizing factor [6], and more recently, relaxin, which is considered an important regulator of renal and cardiovascular function during pregnancy [7], [8]. The serum levels of relaxin increase soon after conception in humans and near day 8 of gestation in rats [9]. Studies by Conrad et al. strongly suggest that relaxin is directly involved in renal vasodilatation and hyperfiltration during pregnancy [7], [10]. Moreover, the chronic administration of relaxin to nonpregnant female and male rats resulted in hyperfiltration [11], [12] and decreased myogenic reactivity of the small renal arteries [13], similar to the changes observed during pregnancy. It has been suggested that the vasodilator actions of relaxin involve the activation of nitric oxide synthase, iNOS [14], and an upregulation of the vasodilatation-inducing endothelin receptor B [15], [16]. Relaxin and relaxin-related peptides interact with four identified receptors. The main relaxin receptor, a leucine-rich repeat G-protein coupled receptor designated LGR7 or RXFP1 [17], is expressed in the aorta and small renal and mesenteric arteries in nonpregnant female and male rats [18]. This suggests a role of LGR7 in arterial function independently of pregnancy. However, to our knowledge, there are no data on the arterial or intrarenal expression level of LGR7 during pregnancy.

In spite of systemic and intrarenal vasodilatation, the plasma level of angiotensin II (Ang II) is increased during pregnancy [19]. This paradox has been attributed to the relative non-reactivity of the pregnant vasculature to vasoconstrictors. It has been well accepted that the increase in vasodilators is involved in the gestational impairment of the myogenic reactivity. On the other hand, Ang II type 2 receptor (AT2) has been shown to display antagonistic effects to those triggered by AT1, including vasodilatation [20], [21]. AT2 is highly expressed during fetal development [22], [23]; however, the role of AT2 in pregnancy-induced vasodilatation or in the reduced vascular reactivity is currently unknown. In order to evaluate the role of relaxin and Ang II receptors in the systemic and intrarenal vasodilatation induced by pregnancy, we measured the mRNA expression levels of AT1, AT2 and LGR7 in the aorta, the renal artery, and the renal cortex.

It has been recognized that intrarenal vasodilatation is the main mechanism responsible for glomerular hyperfiltration in humans and animals [24]. However, it is important to consider that glomerular ultrafiltration is also determined by the glomerular ultrafiltration coefficient (K_f_). Mesangial cells (MCs) are known to influence the glomerular filtration area (and thus K_f_) through their contraction and relaxation in response to vasoactive substances. Importantly, the contraction and relaxation of the MCs are dependent on the intracellular calcium level ([Cai]) [25]. The role of the MCs and the K_f_ in gestational glomerular hyperfiltration is not clear. Based on studies in pregnant rat models, it has been proposed that the K_f_ contributes little to gestational hyperfiltration, since the K_f_ does not change during pregnancy [24], [26]. Since pregnancy is characterized by high levels of circulating Ang II, which is known to reduce the K_f_ by inducing contraction of the MCs, we determined if MCs, cultured from pregnant rats, have a reduced ability to react to Ang II by measuring the changes in [Cai]. We found that MCs from pregnant rats showed a reduced response to Ang II. Therefore, we also evaluated the mRNA expression levels of AT2 and LGR7, as well as inducible nitric oxide synthase (iNOS) in MCs cultured from pregnant rats. Finally, the expression of LGR7 was further analyzed in MCs and kidney tissue from virgin and pregnant rats by immunohistochemistry.

## Results

### Intracellular Calcium

**Figure 1:**
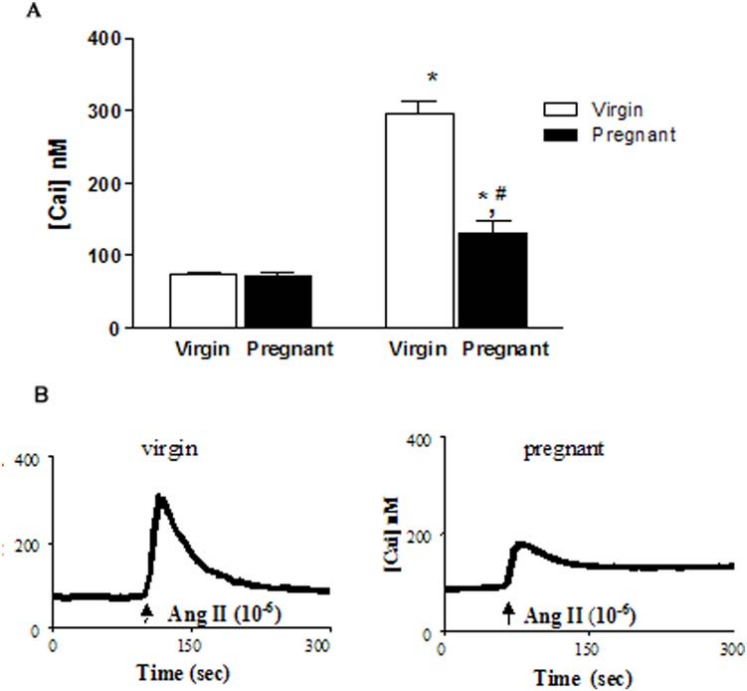
Intracellular calcium concentration ([Cai]) measured under the unstimulated (basal) condition and after the addition of 10^−6^ M Ang II in MCs. A: virgin group (white bars) and pregnant group (black bars). The data bars represent the mean±SEM in MCs isolated from six rats in each group. [Cai] after Ang II stimulation represents the peak value. * p<0.05 vs basal, ^#^ p<0.05 vs virgin. B: Representative traces showing the [Cai] before (basal) and after Ang II (10^−6^ M) addition in MCs isolated from virgin (left panel) and pregnant rats (right panel).

Under unstimulated (basal) conditions, the intracellular calcium concentration ([Cai]) in the MCs did not differ between the virgin and pregnant groups; however, the Ang II-induced increase in the [Cai] was significantly lower in the MCs from the pregnant group compared to the increase in the virgin group (Figure 1A). Figure 1B shows a representative profile of the [Cai] variations in response to Ang II in both groups. As expected, Ang II induced a transient increase in the [Cai] in cells from the virgin rats. Approximately 3–4 minutes following stimulation with Ang II, the [Cai] returned to control levels. In cells from the pregnant group, Ang II induced a peak [Cai] that was clearly lower that in the virgin group, and the [Cai] remained above basal level for at least 5 min.

### Quantitative real-time PCR

**Figure 2:**
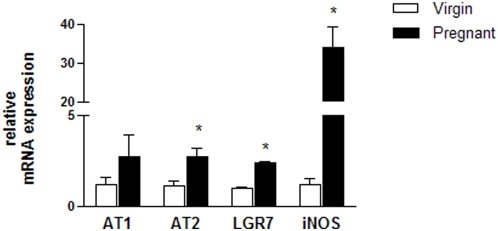
Quantitative real-time RT-PCR for the Ang II receptors (AT1 and AT2), the relaxin receptor (LGR7), and iNOS in MCs isolated from virgin and pregnant rats. Total RNA was isolated from pooled cells that were obtained from three or four culture flasks from each group. Individual samples were normalized to β-actin mRNA levels and the data are expressed as the mRNA expression relative to the virgin group. Values represent the mean±SEM of six samples/group. *p<0.05 vs virgin.

The mRNA expression level of AT1, AT2, LGR7, and iNOS was examined in MCs from virgin and pregnant rats using real-time PCR (Figure 2). The expression of the target mRNA was normalized to the mRNA expression of β-actin for each sample, and the results are expressed as a relative ratio compared to the average of the normal group. The mRNA expression level of all molecules except AT1 was significantly elevated in MCs from the pregnant group, especially the iNOS mRNA expression level, which was 28-fold higher than the virgin group.

**Figure 3:**
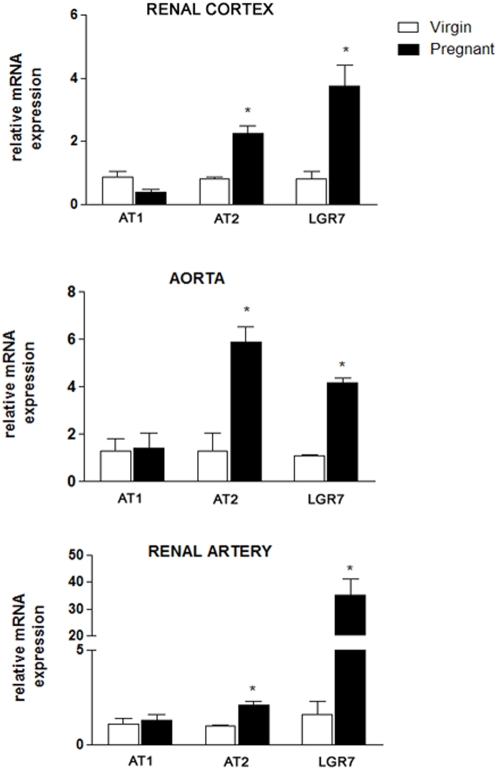
Quantitative real-time RT-PCR for AT1, AT2, and LGR7 in the renal cortex, the aorta, and the renal artery of virgin and pregnant rats. Individual samples were normalized to β-actin mRNA levels and data are expressed as the mRNA expression relative to the virgin group. Values represent the mean±SEM of six samples/group. *p<0.05 vs virgin.

The mRNA expression of the Ang II and relaxin receptors in the renal cortex, the aorta, and the renal artery is shown in Figure 3. The AT1 mRNA expression in the renal cortex (upper panel), aorta (middle panel), or renal artery (lower panel) was not modified by pregnancy; however, there was a significant increase in the mRNA expression of AT2 and LGR7 in all three structures of the pregnant group, including a 30-fold increase in the mRNA expression of LGR7 in the renal artery.

### Immunostaining of LGR7 in the MCs and in the kidney

**Figure 4:**
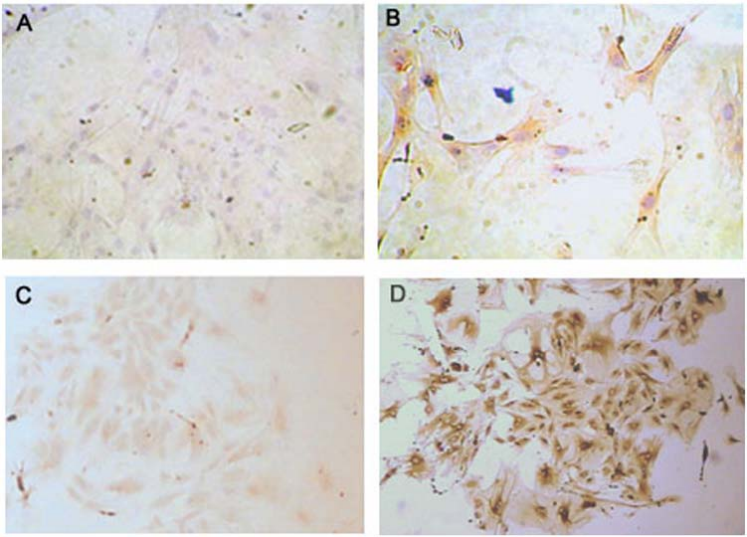
Representative LGR7 immunoassaying (dark brown) of MCs from virgin rats (A) and pregnant rats (B). The omission of primary antibody was the negative control (C) and actin labeling was used as a positive control (D). (200× magnification).

**Figure 5:**
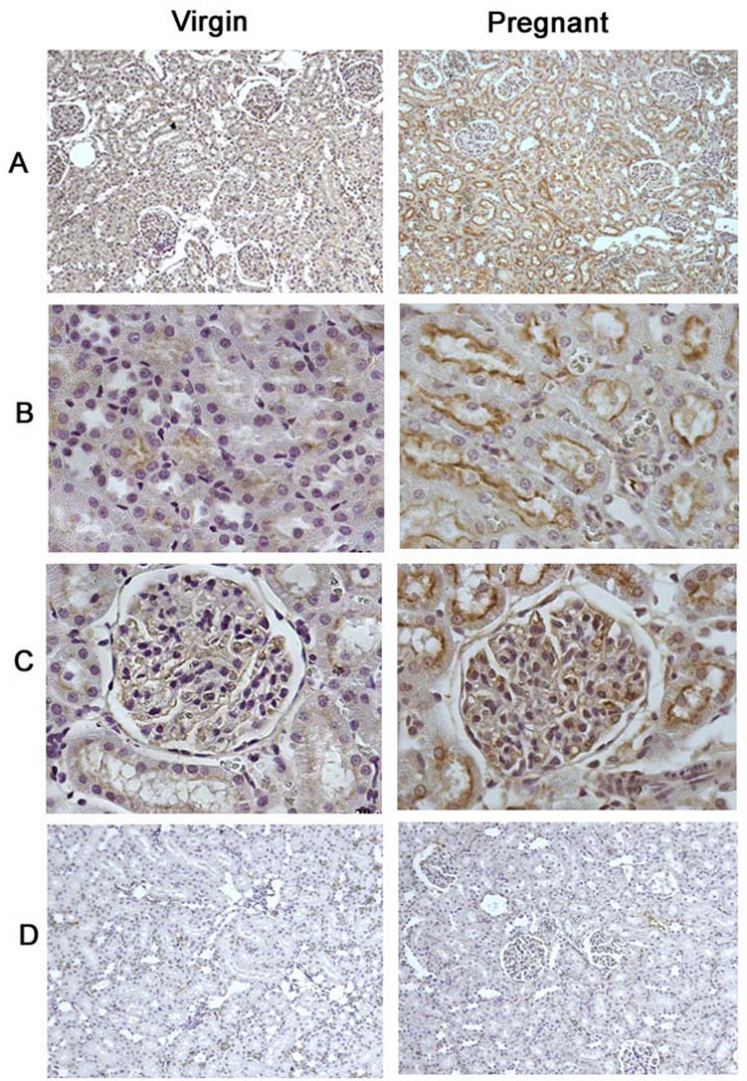
Photomicrograph of kidney tissue from virgin (left panel) and pregnant (right panel) rats. Representative LGR7 immunostaining (dark brown) in the cortex (A, 200× magnification), the cortex showing the apical membrane distribution of LGR7 in proximal tubules (B, 400× magnification), glomeruli (C, 400× magnification) and the negative control (D, omission of primary antibody, 200× magnification).

The LGR7 receptor was expressed abundantly in the MCs from pregnant rats, whereas in the MCs from the virgin group, LGR7 protein was undetectable (Figures 4A and 4B). The distribution of LGR7 in the whole kidney is shown in Figure 5. As seen in a panoramic view of the renal cortex (panel A), the pregnant kidney shows a stronger staining than the virgin kidney. In panel B, the clear distribution of LGR7 in the apical membrane of the tubular cells can be seen. Similarly, the labeling intensity was higher in the pregnant rats compared to the virgin rats. LGR7 staining was also observed in the glomeruli of pregnant rats, as shown in panel C. Panel D shows the negative control.

## Discussion

Due to their contraction/relaxation capacity, which is dependent on the intracellular calcium concentration, MCs constitute an important site of regulation of the K_f_ and thus the single nephron glomerular filtration rate. The present study analyzed the increase in the [Cai] induced by Ang II in MCs cultured from virgin and 12–14-day-pregnant rats. The response of the MCs to Ang II was attenuated in the pregnant group, suggesting a relative insensitivity to this agonist. In spite of the diminished response to Ang II, the increase in [Cai] was sustained in pregnant cells. While we have no explanation at this moment for this behavior, one possible mechanism is that alterations in the IP3 and DAG signaling pathways during pregnancy weaken the regulation of calcium release from the endoplasm reticulum. In support of this, Conrad and co-workers [27] have demonstrated that vascular phosphoinositide metabolism is attenuated in the aortic vasculature of pregnant rats, which could compromise the vascular reactivity during gestation. However, it is not known if this also occurs in the MCs.

The reduced responsiveness of the MCs from pregnant rats to Ang II may be caused by many potential mechanisms, including changes in the expression of the receptors for Ang II and for other vasoactive substances. Actually, the mRNA expression of AT2 was increased in the MCs of the pregnant group, whereas no significant change was found in the mRNA level of AT1. Accordingly, previous studies have demonstrated that pregnancy has little effect on the expression level of AT1 in the vascular vessels, except in the uterine artery [28], [29]. The upregulation of AT2 observed in the present study may minimize the AT1-mediated MC contraction, thereby maintaining the K_f_ during pregnancy, a condition characterized by high levels of Ang II. While this response is probably not enough to induce an increase in the glomerular filtration rate, it may have a permissive role that function in conjunction with the hyperflow-induced hyperfiltration.

We demonstrated that MCs express the relaxin receptor mRNA, although the LGR7 protein level was undetectable in MCs isolated from virgin rats, at least using immunohistochemistry. This suggests that under baseline conditions, there is only a very low level of this receptor expressed. The increase in the mRNA and protein expression of LGR7 in the MCs from the pregnant rats suggests a potential role for relaxin in the modulation of MC function during pregnancy. However, whether the reduced responsiveness of MCs to Ang II during pregnancy is directly or indirectly linked to LGR7 is still unclear and needs further investigation, but one possibility includes the LGR7-mediated increases in NO, as previously demonstrated in vascular smooth muscle cells [30].

It has been shown that iNOS can be induced in MCs by many stimuli, including cytokines and lipopolysaccharides [31], [32]; however, this is the first study evaluating the effect of pregnancy on iNOS gene transcription in these cells. We detected an impressive increase (∼30 fold) in the iNOS mRNA level in MCs from pregnant rats. The stimulus for iNOS synthesis by MCs during pregnancy is currently unknown. Relaxin is a potential candidate, since LGR7 activates nitric oxide synthase in the kidney [12]. Relaxin-induced intrarenal vasodilatation appears to be mediated mainly by eNOS [15], but relaxin is also able to activate iNOS in cultured smooth muscle cells [30]. Therefore, it is reasonable to postulate that relaxin induces NO production by iNOS in MCs during pregnancy. In addition, the stimulation of AT2 increases NO production in the kidney [33], which is mediated by several NOS isoforms including iNOS [34]. The upregulation of AT2, LGR7, and iNOS detected in the present study suggests an involvement of these components in the reduced responsiveness of MCs to Ang II. This reduced responsiveness would help keep the K_f_ constant during pregnancy, even under high levels of circulating Ang II.

Given the vasodilatation properties of AT2 and LGR7, we hypothesized that the expression of these receptors may also be elevated in the vasculature, thereby contributing to the pregnancy-induced changes in the systemic and renal hemodynamics. Our results demonstrated an increase in the mRNA expression of AT2 in the aorta and especially in the renal artery of pregnant rats. There was no change in the expression of AT1. AT2 is highly expressed in fetal development and in some maternal reproductive tissues during pregnancy, including the placenta [35] and the uterine artery [36]. Our results indicate that the upregulation of AT2 during pregnancy is not restricted to the fetal and reproductive tissues but also occurs in the maternal systemic and intrarenal vasculature. Although the measurement of the AT1 and AT2 levels in the present study was performed in non-resistant vessels, it is reasonable to speculate that an increase in the AT2 level may contribute to the maintenance of blood pressure during mid-pregnancy, in spite of the increased levels of circulating Ang II.

The mRNA expression of LGR7 increased 4-fold in the aorta, whereas the increase in the renal artery was approximately 30-fold in pregnant rats, compared to virgin animals. Novak and coworkers [7] demonstrated that the elimination of circulating relaxin by ovariectomy or neutralizing antibody treatment in pregnant rats abolished the gestational elevation in renal perfusion and hyperfiltration but did not prevent the reduction in arterial blood pressure. Taken together, these findings suggest that relaxin plays a key role in the adaptation of the intrarenal vasculature during pregnancy, which involves an upregulation of the relaxin receptor.

The distribution analysis of the relaxin receptor in renal tissue demonstrated that LGR7 is highly expressed on the luminal surface of the proximal tubular cells. LGR7 labeling was much higher in the pregnant kidneys, suggesting a potential role of relaxin in the control of tubular transport during pregnancy. The role of relaxin in water and sodium homeostasis during pregnancy was first proposed by Danielson and co-workers [12]. Also, it has been demonstrated that chronic or acute relaxin infusion resulted in natriuresis and diuresis in rats and healthy volunteers [37], [38]. Taking into account our findings, it is reasonable to hypothesize that relaxin has a direct tubular effect by interacting with LGR7 at the luminal surface and the upregulation of this receptor may regulate tubular sodium reabsorption. Therefore, this study suggests a novel mechanism by which the sodium content and extracellular volume are regulated during pregnancy.

In summary, we provide evidence that the Ang II-induced [Cai] elevation is reduced in MCs from pregnant rats, suggesting a relative insensitivity to this agonist. This response may contribute to the maintenance of the MC tonus, and therefore the K_f_, in the presence of high levels of circulating Ang II during pregnancy. AT2 and LGR7, together with high expression level of iNOS, are potential candidates to mediate this response. The upregulation of AT2 and LGR7 in the aorta and renal artery suggests that Ang II and relaxin may play a role in the systemic and intrarenal vasodilatation observed during normal pregnancy. The presence of the relaxin receptor on the luminal membrane of tubular cells suggests that the interaction of relaxin with its tubular receptors may directly influence tubular transport during pregnancy.

## Materials and Methods

Young virgin female Wistar rats (200–250 g) were obtained from the animal care facility of our Institution (CEDEME-UNIFESP), and the experimental protocol was approved by the Ethical Committee of the Federal University of São Paulo. The animals had free access to standard rat chow and water and were maintained in a temperature-controlled environment (23°C) on a 12 h light/dark cycle. The rats were pair-housed with a male for 2–3 days, and the day when sperm was first detected in the vaginal smears was considered Day 1 of pregnancy (examined daily). Pregnant animals were sacrificed between Day 12–14 of pregnancy, since this period corresponds to the peak of the elevation in the glomerular filtration rate in rats [24]. Age-matched virgin (n = 22) and pregnant (n = 22) rats were anesthetized (pentobarbital), and the kidneys were removed for primary mesangial cell culture. In some animals, the renal cortex, abdominal aorta, and renal arteries were isolated and immediately frozen in liquid nitrogen and stored at −70°C until use. These tissues were used to determine the mRNA expression levels, as described below.

### Primary culture of mesangial cells

The mesangial cells (MCs) were cultured using standard techniques following glomerular isolation by differential sieving [39]. Glomeruli were isolated from freshly removed kidneys from virgin and pregnant rats and then plated at a density of approximately 300 glomeruli/cm into RPMI 1640 supplemented with 20% fetal bovine serum, 50 U/ml penicillin, 2.6 g HEPES acid, and 2 mM glutamine (Sigma Chemical Company, USA). The cells were used between the third and fifth subcultures. The purity of cultures was periodically checked using immunofluorescence, and the cells were negative for human factor VIII antigens and cytokeratin and positive for actin and Thy 1.

### Intracellular calcium measurements

Confluent MCs were resuspended in 2.5 ml Tyrode buffer containing 0.2% bovine serum albumin and incubated in a CO_2_ incubator at 37°C for 30 minutes. The suspension was then centrifuged at 226 g, and the supernatant was aspirated. The pellet was resuspended in 2.5 ml albumin-free Tyrode buffer and transferred to the quartz cuvette of a SPEX fluorimeter (AR CM System, NJ, USA) for autofluorescence determination. The measurements were made with excitation wavelengths of 340 and 380 nm and an emission wave length of 505 nm. The autofluorescence ratio was less than 10%. The MCs were then incubated with 2 µM fura-2/AM and transferred to a Perkin Elmer spectrofluorimeter (LS 5B, Buckinghamshire, UK) to record the fluorescence spectrum of the indicator at an excitation range of 300 to 400 nm and an emission wavelength of 520 nm. The maximum fluorescence of Fura-2AM was observed at 390 nm. The fluorescence peak shifted to 350 nM within a mean period of three hours, indicating the maximum amount of indicator was incorporated into the cell suspension. The cells were washed with Tyrode buffer, resuspended in 2.5 ml Tyrode buffer and transferred to a SPEX fluorimeter programmed for excitation at two wavelengths (340 and 380 nm) with an emission at 505 nm, under constant stirring at 37°C. The first reading of this phase corresponded to the basal value. The cells were then stimulated with 10^−6^ M Ang II [40]. At the end of each experiment, 50 µM digitonin, 1 mM manganese, and 2 mM EGTA were added, and the results are reported as the relative ratio of the 340 and 380 nM excitations, with the reading following the addition of digitonin considered to be 100%. The [Cai] was estimated by the formula of Grynkiewicz [41].

### Quantitative real-time PCR

Total RNA was purified from MCs and tissues (renal cortex, aorta, and renal arteries) using the phenol and guanidine isothiocyanate-cesium chloride method and an appropriate kit (Trizol, Gibco BRL, Rockland, MD, USA). The total RNA (2 µg) was treated with DNase (Promega, Madison, WI, USA) to avoid genomic DNA contamination. The RNA pellet was resuspended in RNase-free water and reverse transcribed into cDNA by the addition of a mixture containing 0.5 mg/ml oligo d(T), 10 mM DTT, 0.5 mM dNTPs (Amersham Pharmacia Biotech, Uppsala, Sweden), and 200 U of reverse transcriptase enzyme (SuperScript RT; Gibco BRL).

The mRNA expression level of the Ang II receptors (AT1 and AT2), the relaxin receptor (LGR7), and the inducible nitric oxide synthase (iNOS) were estimated using real-time RT-PCR and a GeneAmp 5700 System (Applied Biosystems, USA). The cDNA (1 µl) was added to the PCR mixture containing 0.5 mg/ml oligo dT (Amersham Pharmacia Biotech, Sweden), 0.1 M DTT, and 10 mM dNTPs (Amersham Pharmacia Biotech, Sweden). The primers were designed and chosen based on their efficiency and were the following (forward and reverse, respectively): AT1b (5′ atgccagtgtgtttctgctc 3′ and 5′aactcaacactccccattgg 3′), AT2 (5′ cagtggtctgctgggattgc 3′ and 5′ ccatccaggtcagagcatcc 3′), iNOS (5′ aggtgttcagcgtgctccac 3′and 5′ agttcagcttggcggccacc 3′), LGR7 (5′ tgggctcattggccgttctg 3′ and 5′ actccattcgtgccgtagtag 3′), and β-actin (5′ cctctatgccaacacagtgc 3′ and 5′ acatctgctggaaggtggac 3′). The accumulation of the PCR product was monitored in real time using the intercalating dye SYBR Green I (Molecular Probes, USA), which exhibits a higher fluorescence upon the binding of double-strand DNA. The fluorescence for each cycle was quantitatively analyzed using an ABI Prism 7700 Sequence Detection System (Applied Biosystems). A melting curve analysis was performed at the end of each PCR program in order to verify the presence of a single amplification product. The relative gene expression was calculated early in the PCR program, when the amplification curve was logarithmic. The mRNA expression levels were normalized to β-actin expression, and the results are expressed as arbitrary units considering the virgin group as the standard.

### Immunostaining for LGR7 in mesangial cells and kidney tissue

MCs cultured from virgin and pregnant rats were plated on glass coverslips, fixed with 3.5% paraformaldehyde for 45 minutes at 15–20°C, and washed with Tris buffer. The cells were incubated overnight at 4°C with a primary antibody against LGR7 (goat polyclonal IgG, Santa Cruz Biotechnology, CA, USA), at a 1∶80 dilution. The cells were rinsed with Tris buffer and incubated with a biotinylated secondary antibody (1∶50; donkey anti-goat IgG, Santa Cruz Biotechnology) at room temperature for 1 hour. α-Actin (goat polyclonal IgG, Santa Cruz Biotechnology) staining was used as a positive control, and the omission of the primary antibody was used as the negative control. After incubation with the secondary antibody, the cells were rinsed with Tris buffer and then incubated for 30 minutes with horseradish peroxidase–conjugated streptavidin. In order to visualize the labeling, a diaminobenzidine-based solution was used. The cells were counterstained with hematoxylin, and images were captured with a digital camera.

Longitudinal sections from kidneys were fixed in 10% phosphate-buffered formalin, washed with 70% ethanol and embedded in paraffin. Sections (3–4 µm) were cut from the paraffin blocks and counterstained with hematoxylin. The sections were deparaffinized and hydrated in a graded ethanol series. The sections were incubated overnight with a primary antibody against LGR7 at a 1∶75 dilution and then with a biotinylated secondary antibody for 30 minutes at 1∶200 dilution, using a commercial kit (LSAB, Dako, CA, USA). The sections were incubated for 30 minutes with horseradish peroxidase-conjugated streptavidin (LSAB, Dako). In order to visualize the labeling, diaminobenzidine (, Dako, CA, USA) was added for 3–5 minutes.

### Statistical Analysis

The results are presented as the mean±standard error. The data were evaluated using a Student's t-test or Mann-Whitney test, when appropriate. Two-way ANOVA was used to determine the possible interaction between groups concerning [Cai] variation. The level of statistical significance was defined as p<0.05.
